# GETTING TO YES . . . I WILL RESPOND: CHALLENGES AND SUCCESSES SURVEYING AGING SERVICES PROVIDERS TO PRODUCE QUALITY DATA

**DOI:** 10.1093/geroni/igz038.2159

**Published:** 2019-11-08

**Authors:** Lauren Harris-Kojetin

**Affiliations:** National Center for Health Statistics, Hyattsville, Maryland, United States

## Abstract

Voluntary surveys of aging services providers are important data sources for research, quality improvement, and program evaluation efforts to inform evidence-based decision making. Ideally, provider surveys—a type of establishment survey—offer valuable information on providers and services users. However, decreasing survey response rates in recent years raise data quality concerns. This symposium highlights challenges leading to lower response rates (e.g., time constraints, skepticism, confidentiality concerns, getting to the correct respondent); specific data collection techniques tested, what did and did not work, and lessons learned. Although the surveys focus on long-term services and supports (LTSS) providers (e.g., assisted living) and services users (e.g., residents), the session is generalizable to other establishment surveys. Presenters bring extensive survey experience and diverse organizational perspectives—academic research center, national provider association, federal statistical agency, and research contractor. Over the years, the presenters have used their research network to share challenges and lessons learned with each other, which addresses the GSA conference theme, “Strength in Age: Harnessing the Power of Networks.” The first presentation describes test results of a state survey protocol to obtain sampled resident information from assisted living providers. The second presentation examines approaches to increase provider participation in a quality improvement initiative. The third presentation discusses efforts to address response challenges in an on-going national survey of providers in two LTSS sectors. The session allows time for and facilitates interaction with audience members to share their insights and lessons learned.

